# Remote Ischemic Conditioning for Intracerebral Hemorrhage (RICH-1): Rationale and Study Protocol for a Pilot Open-Label Randomized Controlled Trial

**DOI:** 10.3389/fneur.2020.00313

**Published:** 2020-04-28

**Authors:** Wenbo Zhao, Fang Jiang, Sijie Li, Chuanjie Wu, Fei Gu, Quanzhong Zhang, Xinjing Gao, Zongen Gao, Haiqing Song, Yuping Wang, Xunming Ji, the RICH-1 Investigators

**Affiliations:** ^1^Department of Neurology, Xuanwu Hospital, Capital Medical University, Beijing, China; ^2^Beijing Key Laboratory of Hypoxic Conditioning Translational Medicine, Xuanwu Hospital, Capital Medical University, Beijing, China; ^3^Clinical Stroke Research Unit, Xuanwu Hospital, Capital Medical University, Beijing, China; ^4^Department of Neurology, Ningjin County Hospital, Xingtai, China; ^5^Department of Neurosurgery, Heze Municipal Hospital, Heze, China; ^6^Department of Neurosurgery, The Sixth Hospital of Hengshui, Hengshui, China; ^7^Department of Neurology, Shengli Oilfield Central Hospital, Dongying, China; ^8^Department of Neurosurgery, Xuanwu Hospital, Capital Medical University, Beijing, China

**Keywords:** intracerebral hemorrhage, hematoma resolution, remote ischemic conditioning, randomized controlled trial, safety

## Abstract

**Background and rationale:** Although many therapies have been investigated for intracerebral hemorrhage (ICH), none have succeeded in improving the functional outcomes. Remote ischemic conditioning (RIC) has been proven to promote hematoma resolution and improve neurological outcomes in an ICH model; whether it is safe and feasible in patients with ICH remains unknown. This trial aims to assess the safety, feasibility, and preliminary efficacy of RIC in patients with ICH and to plan for a phase-2 study.

**Methods:** A proof-of-concept, assessor-blinded, pilot open-label randomized controlled trial will be carried out with patients with ICH within 24–48 h of ictus. All participants will be randomly allocated to the intervention group and the control group with a 1:1 ratio (*n* = 20) and will be treated with standard managements according to the guidelines. Participants allocated to the intervention group will receive RIC once daily for 7 consecutive days. Cranial computed tomography examinations will be performed at baseline, and on days 3, 7, and 14. Neurological outcomes will be assessed at baseline, and on days 1 to 14, 30, and 90. The primary outcome to be tested is safety. Secondary tested outcomes include changes of hematoma and perihematomal edema volume, incidence of hematoma expansion, functional outcomes, and frequency of adverse events.

**Discussions:** This study will be the first proof-of-concept randomized controlled trial to ascertain the safety, feasibility, and preliminary efficacy of RIC in patients with ICH, results of which will provide parameters for future studies and provide insights into the treatment of ICH.

**Trial Registration:** Clinicaltrials.gov, identifier: NCT03930940.

## Introduction and Rationale

Intracerebral hemorrhage (ICH) is a severe neurological disease and public health issue that accounts for 10–15% of strokes in European and American countries and 20–30% of strokes in Asian countries (1, 2). Although the rate of ICH is far lower than that of ischemic stroke, which is more than 70% of all strokes, its mortality is as high as 50% at 30 days. Furthermore, only 20% of survivors regain functional independence (2). Over recent decades, many corresponding strategies, such as invasive and minimally-invasive hematoma evacuation, blood pressure control, hemostatic therapy, and osmotic treatment, have been investigated; however, few of them have succeeded in improving functional outcomes (3), leading ICH to be the least treatable type of stroke (4), and novel strategies and approaches for the treatment of ICH are urgently needed.

Remote ischemic conditioning (RIC) is a noninvasive systemic protective strategy in which several cycles of brief focal ischemia followed by reperfusion in arms or legs confer protection against more severe injuries in distant organs (5). At present, RIC if applied in arms has been found to benefit patients with acute ischemic stroke, intracranial atherosclerotic stenosis, cerebral small vessel disease, and those undergoing carotid stenting (6–9). Although the underlying mechanisms are not fully understood, current evidence indicates that RIC could reduce inflammation, oxidative stress, and cerebral edema and have other positive impacts (10, 11). Furthermore, in critically ill patients with aneurysmal subarachnoid hemorrhage, RIC applied in the legs has been demonstrated to be feasible, safe, and well-tolerated, with the potential to prevent delayed cerebral artery spasm and to reduce the incidence of both stroke and death (12–14).

In the ICH model, RIC has been found to reduce cerebral edema without exacerbating hematoma size within 72 h of ictus (15, 16). More recently, an experimental study further demonstrated that 6 consecutive days of RIC could improve local cerebral blood flow perfusion, promote hematoma resolution, and improve neurological outcomes (17). However, it remains unknown whether RIC is safe, feasible, and effective in human patients with ICH.

We therefore designed the Remote Ischemic Conditioning for intracerebral Hemorrhage (RICH) study to investigate the safety, feasibility, and efficacy of RIC performed in unilateral arm in patients with ICH. RICH-1 is a pilot study, aiming to determine the safety and feasibility of RIC in patients with ICH and to plan for a prospective phase-2 study (RICH-2) that will investigate the efficacy of RIC.

## Methods

### Study Design

**Figure 1:**
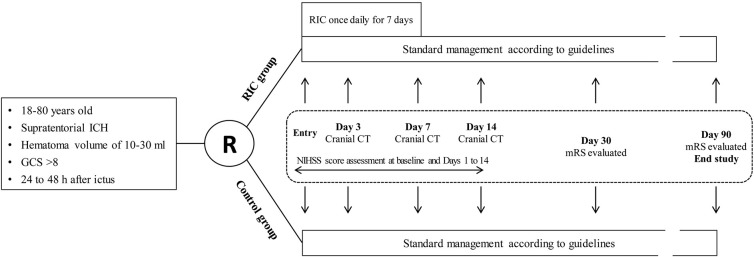
Trial design flowchart. ICH, intracerebral hemorrhage; GCS, Glasgow Coma Scale score; RIC, remote ischemic conditioning; NIHSS, National Institutes of Health Stroke Scale; mRS, modified Rankin Scale; CT, computed tomography.

This is a proof-of-concept, assessor-blinded, pilot open-label randomized controlled trial that will be carried out in four centers with patients who suffered from ICH and will receive nonoperative treatment. The trial design flowchart is illustrated in Figure 1. Participants meeting the inclusion criteria but not the exclusion criteria will be randomly allocated to the RIC group or the control group. Both groups will be treated with the standard management according to the guidelines (4). Remote ischemic conditioning will be performed in the RIC group for 7 consecutive days after enrollment. Cranial computed tomography (CT) examination will be performed at baseline and days 3, 7, and 14. The National Institutes of Health Stroke Scale (NIHSS) score will be assessed by trained investigators blinded to the treatment assignment at baseline and days 1–14 after enrollment. Modified Rankin Scale (mRS) will be evaluated by trained investigators blinded at days 30 and 90.

All participants will be informed about the clinical study and the requirements to give informed consent. This study was approved by the ethical committee of each center and has been registered at *Clinicaltrials.gov* with NCT03930940.

### Patient Population: Inclusion and Exclusion Criteria

Participants will be recruited from the wards. The inclusion criteria are (1) ≥18 and ≤80 years old; (2) diagnosis of supratentorial ICH confirmed by brain CT scan; (3) hematoma volume of 10–30 mL; (4) Glasgow Coma Scale (GCS) score >8; (5) ability to start RIC treatment within 24–48 h of ictus; and (6) informed consent provided by participant or legally authorized representative.

Exclusion criteria are as follows: (1) patients with suspected ICH secondary to tumor, coagulopathy, ruptured aneurysm or arteriovenous malformation, venous sinus thrombosis, cerebral infarction, and traumatic brain injury; (2) ICH concomitant with subarachnoid hemorrhage or intraventricular hemorrhage and planned surgical evacuation for the index hematoma prior to enrollment; (3) evidence of significant shift of midline brain structure (>10 mm) or herniation on brain imaging; (4) known pregnancy or breastfeeding; (5) concurrent participation in another research protocol investigating a different experimental therapy; (6) preexisting neurological deficit (mRS score >1) or psychiatric disease that would confound the neurological or functional evaluations; (7) life expectancy of fewer than 90 days due to comorbid conditions; (8) severe hepatic and renal dysfunction; (9) severe, sustained hypertension (systolic blood pressure >180 mm Hg or diastolic blood pressure >110 mm Hg); (10) contraindication to RIC because of severe soft tissue injury, fracture, or peripheral vascular disease in the upper limbs; (11) any condition which, in the judgment of the investigator, might increase the risk to the patient.

### Randomization

All enrolled participants will be randomly assigned in a 1:1 ratio to the RIC group and the control group (*n* = 20 each). The randomization sequence will be made according to a predefined table generated by a computer program. Subsequently, the randomization sequence that indicated the group allocation will be concealed in sequentially numbered opaque closed envelopes. A research assistant, who will not be involved in the study, will prepare the envelopes prior to the study. After recording baselines measures, participants will be randomly allocated to either the RIC or the control group by the treating physicians who will open the sealed opaque envelopes.

### Interventions

**Figure 2:**
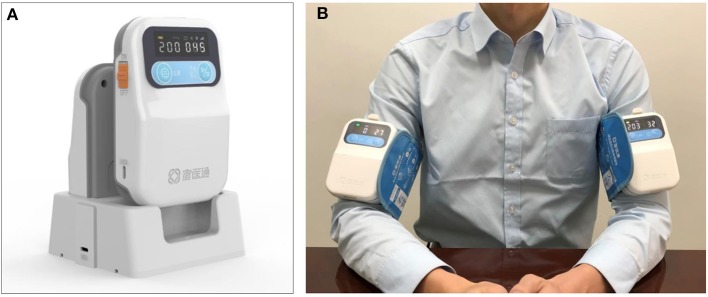
The remote ischemic conditioning device and how it is used. **(A)** The device used for stimulating remote ischemic conditioning. **(B)** Remote ischemic condition performed in human, the device can be used on both sides or one side according to the requirements of the study.

Participants in both groups will receive the standard management according to the guidelines, including blood pressure control, interventions for elevated intracranial pressure and mass effect, treatment of seizures, prevention of venous thromboembolism, maintenance of fluids, treatment of hyperglycemia, rehabilitation, and so on. In addition, participants allocated to the RIC group will undergo the RIC procedure once daily for 7 consecutive days after enrollment, and the interval between two RIC procedure is 24 h. In all the centers, the RIC procedure will be induced by using the same electric autocontrol device with cuffs placed on the nonaffected arm (Figure 2) and will consist of four cycles of arm ischemia with cuffs inflated to a pressure of 200 mm Hg for 5 min followed by reperfusion for another 5 min. The RIC procedure will be performed with assistance from a hospital-based nurse. For participants allocated to the control group, no RIC will be received.

### Outcomes

#### Safety Outcomes Assessment

The safety is defined as any of the following: (1) neurological deterioration defined as an increase of four or more points in the NIHSS or a decrease of two or more points in the GCS score within 7 days of enrollment without a clear explanation; (2) death during the study period regardless of cause; (3) any adverse event that prolongs hospitalizations or requires medical treatment during the study period.

#### Efficacy Outcomes Assessment

The primary efficacy outcome is the change of hematoma volume to be detected on cranial CT images performed at baseline and days 3, 7, and 14. Axial noncontrast CT and images will be obtained at each participant's institution using standard local protocols. Cranial CT scans with 5-mm slice thickness reconstruction will be collected, and the hematoma volume will be measured according to the methods described in a previous study (18).

The secondary outcomes are the following:

1. The change of perihematomal edema volume to be detected on cranial CT images performed at baseline and days 3, 7, and 14: perihematomal edema volume will be measured according to the methods described in a previous study (18).
2. Incidence of hematoma expansion at day 3: hematoma expansion is defined as an increase in the volume of intraparenchymal hemorrhage of ≥33% as measured by image analysis on the 3-days CT scan as compared with the baseline CT scan; the cutoff for hematoma expansion is defined according to the standard set in Brott et al. (19), which is the change in size associated with significant neurological deterioration.
3. Proportion of participants with good functional outcomes at 90 days: functional outcome will be assessed by mRS with a score of 0–3, where higher numbers indicate better functional outcomes.
4. The change of NIHSS score for the first 14 days after enrollment.
5. Frequency of adverse events associated with RIC.

The data of all subjects needed for outcomes measurements will be collected by the treatment physicians or their assistants. Each subject will be marked as a number according to the order they enroll in the trial. All outcomes measurements will be assessed by two observers who are not involved in the development of the clinical treatment plan of subjects and are blind to the treatment assignment; any disagreement will be resolved by reaching a consensus between the two, or if no consensus can be reached, another observer not involved in the development of the clinical treatment plan of subjects and blinded to the treatment assignment will have the final decision. Finally, data of outcomes and information of the group allocation will be collected by an investigator who will perform statistical analysis.

### Data Monitoring

Database management and statistical analyses will be performed by an independent investigator blinded to the treatment assignment of this study. The treatment physicians will monitor compliance with training.

### Sample Size Estimation

When estimating the sample size, no parameters could be referred to, as there is no completed clinical study of RIC in patients with ICH. Fortunately, Dobkin (20) has shown that 15 patients per group are often adequate for determining whether a larger multicenter trial should be conducted. Furthermore, Hertzog (21) has suggested that 10 to 20 patients in each research group are sufficient to evaluate feasibility in a pilot study. Therefore, our goal is to recruit 20 patients in each group. Results of this study should be able to determine the preliminary safety and feasibility of RIC in patients with ICH and will be used to estimate sample size and conduct a power calculation to plan a phase-2 trial.

### Statistical Analyses

All analyses will be done according to the intention-to-treat principle, which will include all participants who enrolled in this trial. Per-protocol analyses, excluding patients who fail to complete the follow-up, will be managed as a supplement of the intention-to-treat analysis to further confirm the results. If the between-group difference in the change of hematoma volume at day 7 is not <12% in favor of RIC, we would move forward with a phase-2 trial.

Categorical variables including the incidence of hematoma expansion, the proportion of good functional outcomes, and the frequency of adverse events will be presented as counts and percentages. These will then be analyzed with χ^2^ test, Fisher exact test, or continuity correction where appropriate. Continuous variables including NIHSS score, GCS score, and hematoma and perihematomal edema volume will be presented as mean and standard deviation or median and interquartile range. These will be analyzed with independent *t-*test or rank-sum test where appropriate. For missing data, we will conduct a sensitivity analysis and use multiple imputations to impute values for those with missing data as continuous data; for clinical events (e.g., death), we will regard the patients lost to follow-up as nonevents in both groups.

The statistical analyses will be conducted with SPSS statistics software for Windows version 20.0 (Armonk, NY, IBM Inc.).

## Discussion

Recently, the Minimally Invasive Surgery Plus Alteplase for Intracerebral Hemorrhage Evacuation III trial that evaluated the efficacy of minimally invasive evacuation followed by thrombolysis in ICH patients has completed, but it failed to improve functional outcomes despite a significantly decreased hematoma volume (22). Therefore, exogenous removal of hematoma may not be as effective as expected. Fortunately, the Intracerebral Haemorrhage Deferoxamine trial found that deferoxamine was safe in patients with ICH and merited further investigation in a phase 3 trial (23). Therefore, strategies that promote hematoma resolution and inhibit the secondary injury of hematoma appear to be promising in the future.

Remote ischemic conditioning has been found to attenuate perihematomal edema, improve cerebral blood flow, and promote hematoma resolution in ICH model (15, 17). In this study, the safety and feasibility of RIC will be determined, and cranial CT will be performed at days 3, 7, and 14 to ascertain the potential effects of RIC on hematoma resolution and perihematomal edema, which will provide parameters for the design of a phase-2 study. Approximately 40% of patients experience hematoma expansion over the first 24 h after ictus (19), which further exacerbates the damage from ICH and is associated with neurological deterioration and worse outcomes (24). In this study, we exclude patients within 24 h of ictus and recruit patients at 24 to 48 h. One reason for this is to ensure safe implementation of the present study. Another reason is that the aim of the RICH trial is to evaluate the effects of RIC on hematoma resolution and edema instead of hematoma expansion. In addition, two other ongoing clinical trials (RESIST, NCT 03481777; SERIC-sICH, NCT03484936) are recruiting ICH patients within 4 and 12 h of ictus respectively and evaluating the effects of RIC on hematoma expansion.

There are limitations to this study. First, the RIC dose that will be used in this study is rather pragmatic and tailored to ICH patients, but it may not be the optimal one. In addition, a sham procedure is not planned in this study, and the participants will be not blinded; this may bias the study results. To alleviate potential bias, all outcomes raters will be blinded to the treatment assignment of this study.

In conclusion, the RICH-1 study is designed to identify the feasibility and safety of RIC in patients with ICH. Additionally, the preliminary efficacy results will provide parameters for the design of a future phase-2 trial.

## Ethics Statement

The study protocol was reviewed and approved by the ethic committee of each center involved. The participants will provide their written informed consent to participate in the study.

## Author Contributions

XJ conceptualize the study and contribute to the study design and implementation as Principal Investigator. FJ and SL make substantial contributions to the design and content of the trial. ZG, HS, and YW contribute to the design of the trial from their area of expertise. CW, FG, QZ, and XG collaborate in the implementation of specific procedures. WZ contributed to the design, implementation, and writing of the protocol. All authors reviewed the manuscript and provided the final approval for the manuscript.

## Conflict of Interest

The authors declare that the research was conducted in the absence of any commercial or financial relationships that could be construed as a potential conflict of interest.
